# How Does Survey Timing Influence Apparent Wasting Trends? A Case Study from Senegal

**DOI:** 10.1016/j.cdnut.2024.104480

**Published:** 2024-10-18

**Authors:** Karan S Shakya, Leah Bevis, Rebecca A Heidkamp, Andrew L Thorne-Lyman

**Affiliations:** 1 Department of Agricultural, Environmental and Development Economics, Ohio State University, Columbus, OH; 2 Center for Human Nutrition, Department of International Health, Johns Hopkins Bloomberg School of Public Health, Baltimore, MD

**Keywords:** wasting, tracking, progress, sustainable development goals, Senegal

## Abstract

**Background:**

Child wasting is known to exhibit seasonal patterns, but few studies have examined how the seasonality of wasting affects tracking of wasting trends over multiyear periods.

**Objectives:**

We explored the seasonality of wasting in Senegal relative to multiyear changes and examined implications for tracking. We tested whether month-fixed effects reduced bias in estimating longer-term wasting trends given variation in survey timing.

**Methods:**

The average prevalence of child wasting (weight-for-height *z*-score < −2) and 95% confidence intervals were calculated by month and year from the continuous Demographic and Health Surveys (DHS) from 2012–2019. Peak and low wasting season estimates were defined as the 4 highest and the 4 lowest months of average wasting prevalence. Month-adjusted annual wasting estimates were generated using month-fixed effects linear regression, and the effectiveness of this method of bias adjustment was examined in simulated datasets.

**Results:**

Nationally, wasting fluctuated from 2013–2019, with the lowest annual prevalence of 6.0% (95% confidence interval [CI]: 5.1, 7.0%) recorded in 2014 and the highest in 2017 at 9.0% (95% CI: 8.3, 9.8%). Pooled across years, the peak wasting season prevalence was 8.8% (95% CI: 8.3, 9.3%), and low wasting season prevalence was 6.4% (95% CI: 5.7, 7.1%). Month-adjusted wasting estimates did not differ notably from raw wasting prevalence estimates. Simulations demonstrated that adjusting for months reduces bias in wasting when surveys are conducted 1 or 2 mo apart across waves but fails to reliably do so when surveys are conducted in different seasons across waves.

**Conclusions:**

Seasonal fluctuations in the prevalence of wasting can be large enough to bias the interpretation of multiyear trends. Efforts should be made to conduct national surveys at the same time of year. Seasonality adjustment using month-fixed effects works more reliably when the differences in survey periods across waves are minimal.

## Introduction

The prevalence of wasting among children aged <5 y reflects relatively recent exposure to shocks including caloric deficits, poor food quality, and infectious diseases and is strongly associated with the risk of child mortality [1,2]. Wasting is now receiving greater attention outside of humanitarian emergencies due in part to the realization that many countries have a high prevalence of wasting that contributes to under-5 mortality rates [3]. The 2012 World Health Assembly set a target of reducing wasting to <5% by 2025, and the Sustainable Development Goals aim to eliminate it completely by 2030 [4,5]. In most countries the main data sources for tracking national wasting progress are large scale national surveys such as the Demographic and Health Surveys (DHS) and the Multiple Indicator Cluster Surveys (MICS), although some countries also collect national wasting data through Standardized Monitoring and Assessment of Relief and Transitions (SMART) surveys and ad hoc nutrition surveys. Tracking efforts such as the Global Target 2025 Tracking tool [6] and the Global Nutrition Report Country Profiles [7] consolidate prevalence data from multiple surveys over time and estimate trends to track progress toward global and national goals.

A challenge to tracking progress toward global and national goals is that wasting often exhibits strongly seasonal patterns. Nationally representative surveys such as the DHS and the MICS are not explicitly designed to factor seasonality into the indicators they generate. In many countries, the national prevalence of wasting is only measured every few years and not always in the same season [8,9]. Data collection for a given survey can even span >1 season. This challenge is explained on the Global Sustainability Development Goal Indicator Platform, “surveys are carried out in a specific period of the year, usually over a few months. However, this indicator can be affected by seasonality… Hence, country-year estimates may not necessarily be comparable over time” [10]. Similarly, the 2023 Joint Child Malnutrition report noted that “The lack of methods to account for seasonality and incident cases of wasting and severe wasting hampers the interpretability of annual trends” and stated that the presented wasting estimates were not adjusted for seasonality [11].

Most of the peer-reviewed literature on the seasonality of wasting has focused on identifying seasonal drivers of wasting including infectious disease, cropping cycles, precipitation, and temperature [[12], [13], [14], [15], [16], [17], [18], [19]]. In comparison, although some articles have described the problem that seasonality introduces into interpretation of wasting trends over time, few articles have sought to quantify the magnitude of bias that seasonality may introduce to estimate the change in wasting prevalence over multiyear periods or to correct for it. One of these, from India, sought to understand how differences in the timing (month) and location of measurements in national surveys spanning the period from 2005–2006 to 2015–2016 may have biased wasting and stunting trends over the period between the 2 surveys [20]. Month and state fixed effects were used in a regression framework to adjust for differences in the month and state of data collection of the 2 surveys. Another article from India sought to develop a more general solution by pooling together data from 4 large national surveys over time to develop regression models that could characterize the nature of seasonal trends in wasting [8]. However, although many articles have described the seasonality of wasting in various African countries [9,[12], [13], [14], [15], [16], [17], [18]], to our knowledge, none have sought to understand how it may affect longer-term estimates or to test approaches to mitigate bias that seasonality may introduce to the interpretation of longer-term trends.

Using data from the nationally representative continuous DHS conducted in Senegal from 2012 to 2019, our study aims were to:

1) Examine the magnitude of seasonality in wasting prevalence in Senegal nationally and among rural and urban households in relation to longer-term trends.
2) Develop annual estimates of wasting in Senegal that account for differences in the month of data collection across survey years by using month-fixed effects in a linear regression framework.
3) Using simulated datasets based on data from Senegal, explore the effectiveness of month-fixed effects as an approach to reduce bias introduced by variation in the timing of surveys used to estimate longer-term trends.

## Methods

### Data

Data from the continuous DHS from 2012 to 2019 were accessed from the DHS Program website [21]. A stratified 2-stage cluster design was employed by the DHS, where enumeration areas were initially collected from the census. Then, in the second stage, a sample of households was drawn from each enumeration area. The sample was nationally representative for all years and stratified by rural/urban areas within the 14 regions of Senegal. The Children’s Recode, which contains information on children’s anthropometric outcomes, was used. Our analysis incorporated the 2-stage stratified sampling design and accounted for any changes in the survey strata by adjusting for year-specific stratified child survey weights. The number of survey strata for Senegal’s DHS changed in 2013, and year-specific stratified household survey weights were used to account for these changes.

A novel feature of the Senegal continuous DHS is that the survey was conducted annually, with 2 data collection periods that together span ∼9 mo. This contrasts with the conventional DHS, which are typically conducted once every 5 y within a single survey period lasting ∼4 mo (sometimes longer in large countries). Data from 2012 and 2013 were combined into a single year for the purposes of this analysis and are labeled 2013 because they were part of the same survey cycle. Sample sizes across years and months are listed in Supplemental Table 1. Despite this DHS survey taking place every year, surveys were not conducted every month, necessitating the pooling of data from all years to obtain a comprehensive sample of children for each month to study seasonality in child health outcomes. Surveys were least likely to be conducted in December, January, and February and were most likely to be conducted during May through October. Monthly wasting prevalence across years is presented in Supplemental Figure 1, with the wide confidence intervals (CIs) supporting the value of pooling sample observations to identify a clear seasonal pattern for wasting in Senegal.

### Outcome definition

Wasting was defined as a child’s weight-for-height *z*-score (WHZ) <2 standard deviations of the World Health Organization growth standards [6]. Consistent with the DHS survey design, WHZ values < or >5 standard deviations were flagged as invalid observations and omitted from our analysis. From our sample of 52,393 children, ∼11.5% were excluded due to invalid or (more frequently) missing observations (Supplemental Table 2).

### Analysis

All estimates were calculated as weighted averages according to the 2-stage sampling design. Our adjustment follows DHS protocol for pooled cross-sectional analysis, accounting for changes in both survey strata and probability sampling units over the years. The resulting survey weights reflect the probability of being sampled in any given year, allowing averages to retain their nationally representative status.

First, the temporal trends in wasting were analyzed. The prevalence of wasting and 95% CIs were estimated over years, over months, and across rural and urban households. Stratification by rural and urban regions was chosen because the DHS surveys are representative of these regions. Based on the monthly trends observed, the maximum seasonal contrast in wasting was examined by comparing 2 extreme periods in a year; 4-mo periods were chosen to approximate the duration of many DHS and MICS surveys. “Peak wasting season” was thus defined as the 4 consecutive months with the highest average prevalence (July to October), and “low wasting season” was defined as the 4 consecutive months with the lowest average wasting prevalence (January to April). The definitions for the periods of peak and low wasting were driven by data rather than previous observations of seasonality from the region, but closely align with the periods before and after the harvest season in this context. The prevalence of wasting during these 2 extreme time periods was estimated for subgroups of rural and urban households because policies are often made based on rural and urban differences.

Second, using a similar approach to research undertaken in India [8,20], we estimated the “month-adjusted” wasting prevalence in each year by using a linear regression framework to control for month-fixed effects. Since the timing of surveys varied slightly over the years, it seemed plausible that adjusting for the month of the survey might yield trends in wasting that are less biased (closer to the truth) than the trends observed in raw yearly means. However, this month-fixed approach may have been less effective in certain data scenarios, such as when there is limited seasonal overlap across data rounds, or few years of data are available. Therefore, the reliability of this method remains unclear.

To address this gap, we explored the effectiveness of this method using simulated data. The objective of this simulation exercise was to determine the degree to which using linear regression with month-fixed effects corrects for bias due to seasonality under different scenarios of survey timing and duration. First, we created a simulated dataset using 500 observations for each month of the 7-y observation period. A wasting indicator was created that mimicked the seasonal variation in wasting rates observed in Senegal but with slight variation (noise) purposively introduced to those seasonal patterns through random variation. No year-to-year variation was created in wasting rates.

To explore how well the fixed effects approach works in conditions similar to the Senegal continuous DHS (which collects data for 9 mo of the year), we sampled 9-mo periods randomly for each of the 7 y. In a second simulation exercise, we sampled 4-mo periods from each year, under 3 scenarios: *1*) all survey periods were sampled from the first half of each year (representing a near-ideal scenario in which survey periods are mostly aligned across years); *2*) a scenario in which 4 of those survey periods were fixed, but 3 survey periods were sampled to begin as late as May to July (representing a less successful sampling scenario, where 3 surveys were conducted later); and *3*) those same 4 survey periods were fixed, but 3 survey periods were sampled to begin as late as August to September (representing a scenario with poor alignment, where 3 surveys were conducted very late, in a totally different season). Once the simulated datasets were created, month-adjusted wasting trends from simulated datasets were compared to the “true” wasting trends in the simulated datasets that spanned all months and all years.

Data were analyzed using STATA 17.0 (Stata Statistical Software, Release 17, StataCorp LLP). Ethical oversight of the Senegal DHS is managed by the institutional review board at ICF International. The use of these publicly available deidentified datasets is not considered human subjects research by the institutional review boards of the Ohio State University or Johns Hopkins Bloomberg School of Public Health.

## Results

Anthropometric and other characteristics outcomes of children in the sample are presented in Table 1. About 70% of observations were children from rural areas, which had a higher wasting prevalence (8.8%) than urban areas (6.3%). The prevalence of stunting was also considerably higher among rural children (21.4%) than urban (12.1%).

**TABLE 1:** Characteristics of the pooled data set and rural/urban subpopulations.

	Rural	Urban	National
Number of children aged <59 mo	32,326	13,811	46,137
WHZ (mean *z*-score)	−0.6 (1.08)	−0.42 (1.1)	−0.54 (1.09)
Wasting (% with WHZ <−2)	8.8	6.3	7.9
HAZ (mean *z*-score)	−1.08 (1.26)	−0.65 (1.21)	−0.92 (1.26)
Stunting (% with HAZ <−2)	21.4	12.1	17.9

All statistics are across sample children, not across households.

Standard deviations in parentheses. All estimates are weighted.

Abbreviations: HAZ, height-for-age *z*-score; WHZ, weight-for-height *z*-score.

**FIGURE 1:**
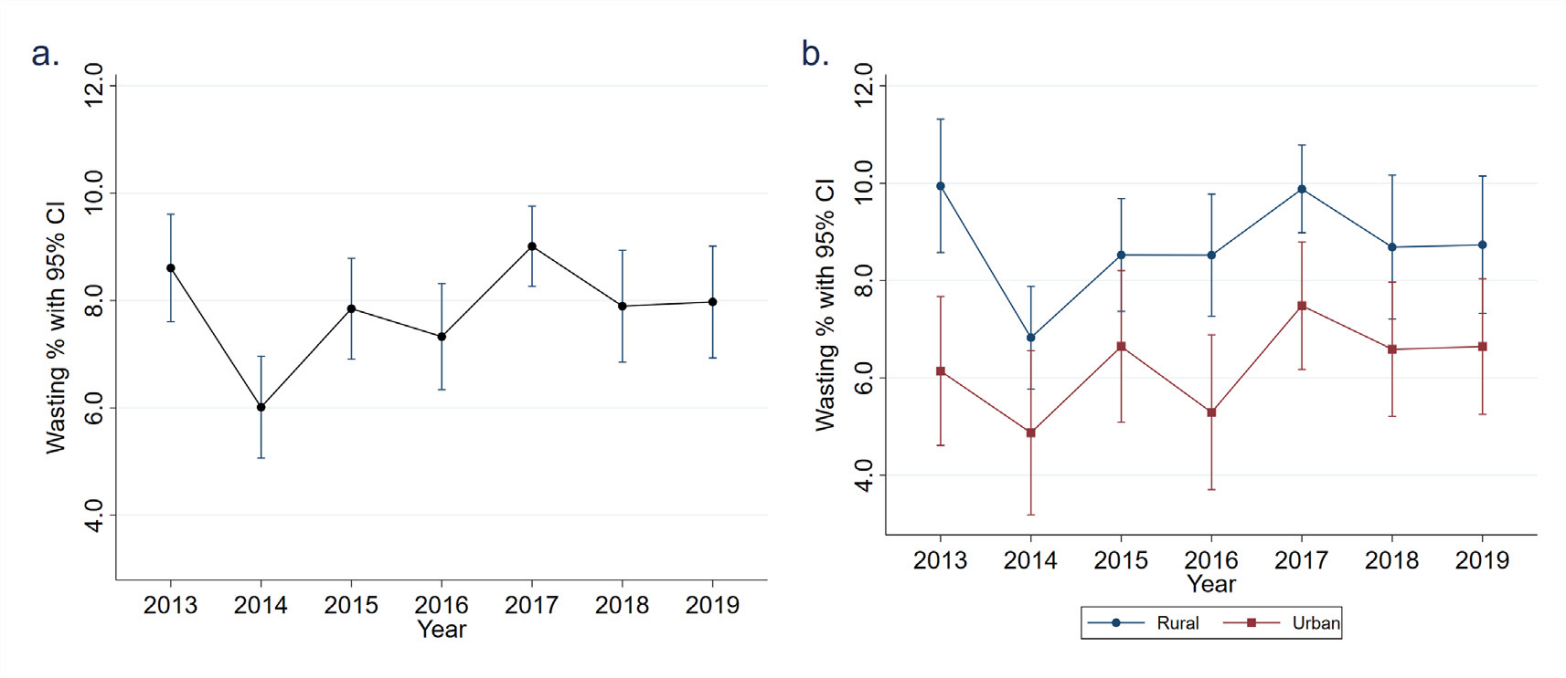
Wasting prevalence (A) by survey years and (B) across rural and urban households. CI, confidence interval.

The annual prevalence of wasting over time fluctuated considerably from 2013 to 2019, with 2014 marking the lowest annual prevalence of 6.0% (95% CI: 5.1, 7.0%) and 2017 marking the highest at 9.0% (95% CI: 8.3, 9.8%) (Figure 1A). From 2014 to 2019, the national prevalence of wasting increased by roughly 2%. The prevalence was higher in rural areas than in urban areas by ∼2% with parallel trends and fluctuations observed in both areas over time (Figure 1B). Rural areas had the highest wasting of 9.9% (95% CI: 8.6, 11.3%) in 2013 whereas urban areas had the highest wasting of 7.5% (95% CI: 6.2, 8.8%) in 2017.

**FIGURE 2:**
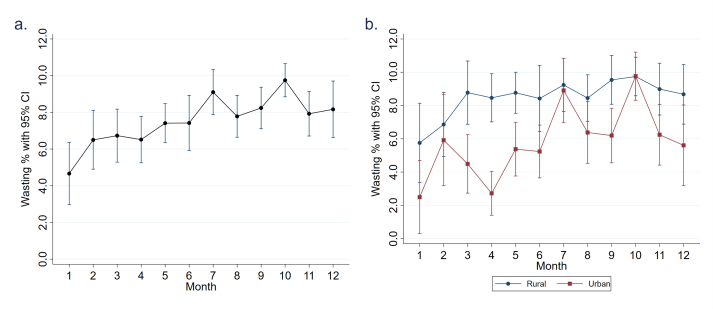
Wasting prevalence estimates pooled across survey years by (A) months and (B) months disaggregated for rural and urban households. CI, confidence interval.

The average prevalence of wasting by month, averaged across years, was highest in October at 9.8% (95% CI: 8.8, 10.7%) with a second peak observed in July at 9.1% (95% CI: 7.9, 10.3%) (Figure 2A). The lowest prevalence of wasting was observed in January. Wasting was consistently higher in rural households than in urban households, except in July and October when urban wasting rates rose to meet rural wasting rates (Figure 2B). Seasonal fluctuations were more pronounced for urban households, with the average wasting prevalence fluctuating across a range of ∼7.3% points compared to rural households, which had fluctuations of 3.9% points. The 2-peak pattern in wasting prevalence was driven by urban households; in rural households, a more gradual increase in wasting rates resulted in a single peak in October.

**FIGURE 3:**
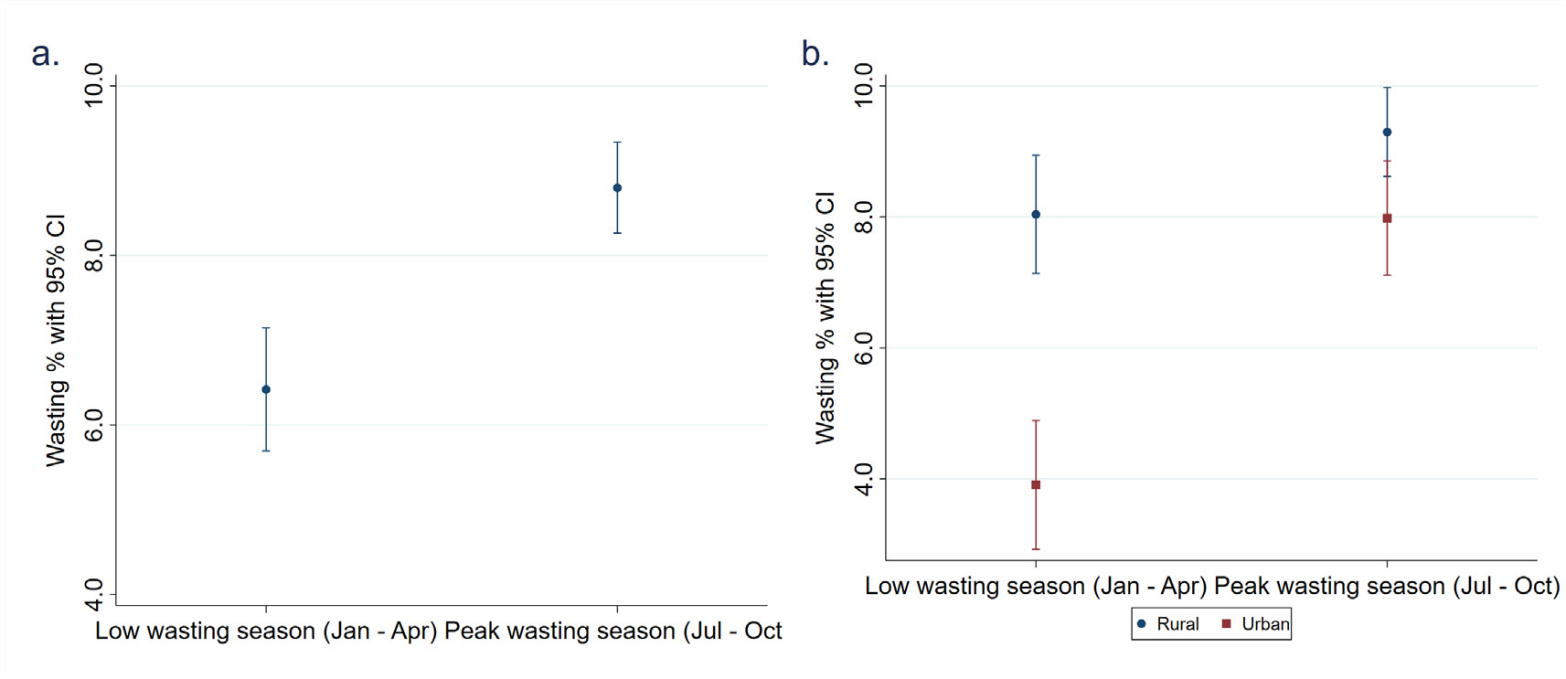
Wasting prevalence estimates pooled across survey years by (A) wasting seasons and (B) wasting seasons disaggregated for rural and urban households. Apr, April; CI, confidence interval; Jan, January; Jul, July; Oct, October.

The national prevalence of wasting for the 4-mo peak wasting season of 8.8% (95% CI: 8.3., 9.3%) was higher than the prevalence of wasting for the 4-mo low wasting season 6.4% (95% CI: 5.7, 7.1%) (Figure 3A). For rural households, the difference in wasting prevalence across the 2 extreme seasons only differed by an average of 1.3% points, whereas a larger average difference of 4.1% points was observed for urban households (Figure 3B). These differences in the wasting prevalence between these 2 extreme seasons in a single year for both rural and urban households was large relative to the differences observed in wasting prevalence across years (3.0% points at most at the national level).

**FIGURE 4:**
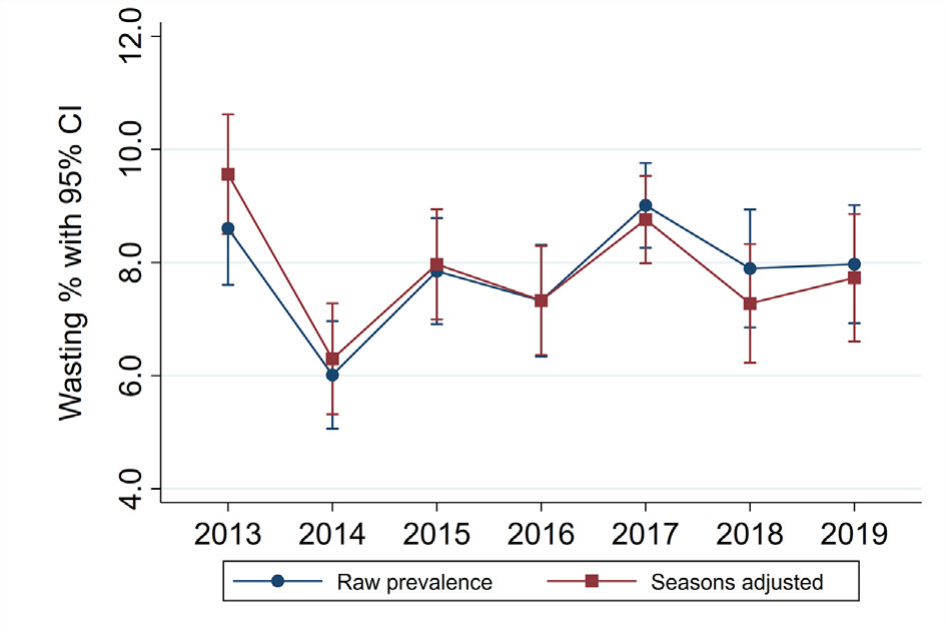
Raw wasting prevalence compared with month-adjusted wasting prevalence by year. CI, confidence interval.

Raw annual prevalence of wasting compared with prevalence estimates adjusted for survey season using month-fixed effects is presented in Figure 4. The 2 sets of yearly wasting prevalence estimates appeared similar, although from 2014 onwards, the month-adjusted estimates were more stable across time than the raw estimates. The regressions used to graph this figure are presented in Supplemental Table 3.

**FIGURE 5:**
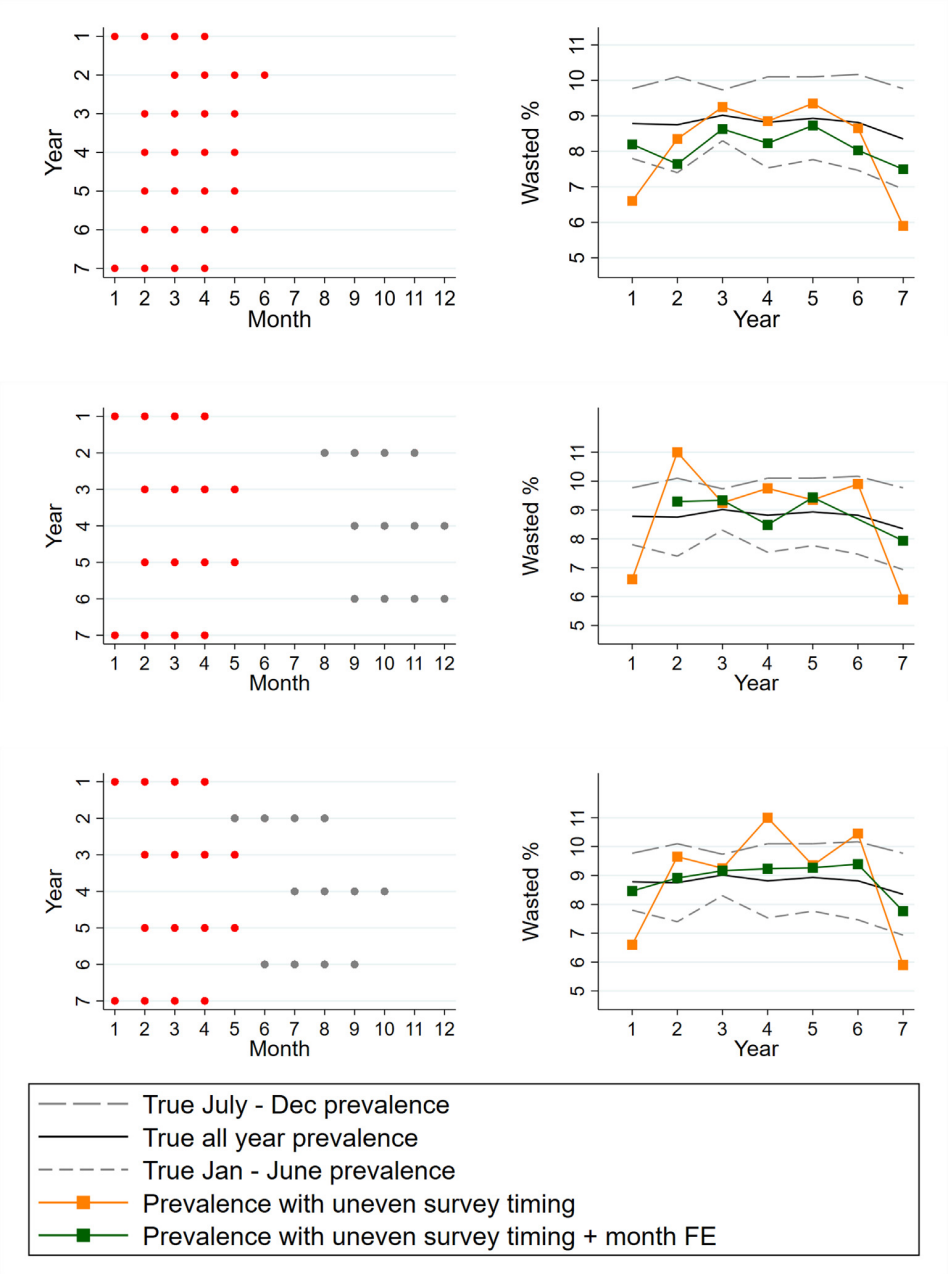
Simulated, true wasting prevalences in each year compared to estimated wasting prevalence with and without month-fixed effects in three sampling scenarios: (A) all seven surveys conducted within the first half of each year; (B) three surveys beginning as late as May–July; (C) three surveys beginning as late as August–September. Dec, December; FE, fixed effects; Jan, January.

Although month-fixed effects usually reduce bias in simulated datasets that mirror the 9-mo sampling period of the Continuous DHS survey in Senegal (Supplemental Figure 2), they do not always do so in simulated datasets that mirror the 4-mo sampling period of most DHS and MICS surveys (Figure 5). For each panel, the left-hand side of Figure 5 illustrates survey timing by year, and the right-hand side illustrates true yearly wasting prevalence (for the entire year and for the first and second half of the year) and estimated wasting prevalence with and without month-fixed effects under each survey timing scenario. In Figure 5A, we observed that when all surveys were timed similarly (i.e., in the first half of the year), adjusting for month improved wasting estimates to be closer to the true January to June wasting estimate, especially in the years when survey timing differed the most (i.e., in years 1 and 7). In Figure 5B, the 4-mo survey periods varied more across the year, and month-adjusted estimates again reduced bias due to uneven survey timing (e.g., are higher than raw estimates in years 1 and 7 and lower in years 4 and 6). In this data scenario, adjusted wasting estimates are closest to the true, year-round prevalence, though not as close as Figure 5A adjusted estimates were to the early-year truth.

In Figure 5C, the 4-mo survey periods varied most dramatically across the year, which caused month and year to become collinear, and one adjusted estimate (for year 1) was lost. Adjusted wasting estimates no longer seemed to be reducing bias in this scenario and were no more stable than the raw estimates. In fact, bias (vis-à-vis all-year truth) in raw estimates and month-adjusted estimates was inversely correlated. Notably, not all simulated datasets showed month adjustment to be ineffective when survey month varied dramatically over the year; Supplemental Figure 3C demonstrates a scenario where month-adjusted prevalence estimates closely mirrored the true, all-year prevalence trends. Supplemental Figure 3A and B also show effective bias reduction under month-fixed effects. These 2 examples were chosen to demonstrate the variation in effect of adjusting for month when estimating seasonal wasting prevalence.

## Discussion

With many countries investing significant resources to prevent and treat wasting over multiyear periods, it is important that global monitoring systems optimize measurement approaches to minimize the effects of seasonal bias on estimates of longer-term tracking of wasting [8]. We investigated the magnitude of bias that seasonality might introduce into measurement of multiyear trends using data from the continuous DHS in Senegal (2013–2019). Nationally, within these years, the point estimate of prevalence fluctuated within a range of ∼3%. Due to the frequent data collection of the continuous DHS, a low and high wasting season could be defined, revealing that national wasting fluctuated by 2.4% points across these seasons. These findings suggest that in this context, seasonal wasting patterns are strong enough to introduce bias into multiyear estimates and suggest the need to be vigilant during survey design, data analysis, and interpretation of results to reduce the effects of seasonal bias on trends of wasting over time.

Seasonal variation in wasting could also be observed in urban areas, which include small towns in Senegal’s DHS data. Although this finding may initially appear surprising, seasonal variation in climactic conditions seems to drive child wasting in Senegal largely through impacts on malaria and water-borne illness [22], both of which may still impact child health in Senegal’s small towns. Additionally, food prices vary seasonally in African towns and cities [[23], [24], [25]], with near-certain impacts on urban food security and child nutritional status.

Frequent data collection across months every year made the continuous DHS for Senegal close to comparable in each year in terms of survey timing. For that reason, adjusting for the month of data collection in our wasting prevalence estimates did not result in significant changes to the raw, year-specific wasting values. The most notable difference was in 2013, where the month-adjusted wasting value was roughly 1% higher than the raw wasting values, and in 2018, where the month-adjusted wasting value was 0.5% lower than the raw wasting values. However, in all years, the 95% CI of both the month-adjusted and raw wasting values overlapped.

The simulations we ran demonstrated that month-fixed effects in a regression framework are likely to improve estimates of yearly wasting prevalence (i.e., reduce bias due to seasonal variation in survey timing) where the duration of data collection is quite long, such as the Senegal DHS, which averaged 9 mo. However, the performance of this correction approach was more variable with shorter 4-mo surveys. When shorter surveys are all conducted within 1 period of the year (as in Figure 5A), adjusting for month may still do well reducing bias—that is, month-adjusted wasting estimates will likely be closer than raw estimates to the true wasting prevalence for that period of the year. However, when survey timing varies dramatically across survey years, month-adjusted estimates are less reliable (less likely to reduce bias) and less interpretable (because it is unclear which part of the year adjusted wasting estimates would represent). Also, in some cases when survey periods are highly dispersed, month- and year-fixed effects become collinear and so one or multiple year-fixed effects must be dropped from the regression, resulting in adjusted prevalence rates for only a subset of years.

The challenge of tracking wasting trends over multiyear periods, given the seasonality of wasting, has been previously noted in the peer-reviewed literature [8]. Many tracking tools also provide cautionary footnotes as part of data presentation, although the season or dates of data collection are not often presented by such tools. Data collection systems such as the DHS and MICS often strive to collect data at the same time of year, when possible, but do not always succeed. A tabulation of DHS survey timings for other Sub-Saharan countries neighboring Senegal shows that these surveys are carried out during different seasons in different years, in each country (Supplemental Table 4). Because these survey periods are fairly short (<half the year) and vary dramatically across a small sample of years, it seems unlikely that adjusting for month-fixed effects in a linear regression setting would reduce seasonal bias in wasting estimates. Collinearity between years and months might also lead to a reduced number of year-specific wasting estimates. Standalone national nutrition surveys such as SMART surveys also have a shorter data collection period than the DHS, which would be expected to lead to even greater seasonal bias in trends if not collected in the same season over time.

Although we are not aware of other studies that specifically examine the challenge of how seasonal bias affects the tracking of annual wasting estimates over time in the Sahel, the seasonality of wasting has been studied in the Sahel [14,15,18] and in the Greater Horn of Africa [13] as well as other countries in Africa [12,16]. In India, researchers found that an increase in wasting prevalence over time was at least partially explained by different timing of surveys and variability in the sampling of geographic locations [8]. The magnitude of seasonal wasting differences that we observed in Senegal is similar to observations from Greater Darfur of ∼1.8% points [26]. A meta-analysis of surveys from the Greater Horn of Africa found that hunger season was associated with an average wasting estimate 1.3% points higher than in postharvest, but also showed substantial variation, ranging from 4.4% points lower in Somalia to 4.8% points higher in Sudan after adjusting for many factors [13]. The substantial variation in patterns of wasting by country and even within country suggests the complexity of the task of correcting for seasonality, which requires estimating both the timing and magnitude of seasonal patterns in wasting [12]. Planning for same-season data collection is a simpler solution, although it has been difficult to achieve this historically in many countries due to competing factors that influence survey planning.

A key strength of this study is that the estimates reflect multiple years of data collection at the national level in Senegal using the same DHS measurement and sampling that many countries use to generate national estimates of wasting. An important limitation to note, despite having observations for several months in different years, data was quite sparse for certain months, particularly January, February, and December. This resulted in a disjunction between January and December estimates. Furthermore, these data gaps prevented us from calculating the *true* unbiased prevalence of wasting. To make such a claim, uniform observations across every month for each year would be necessary. This, however, is infeasible for any survey aiming to collect frequent data at a nationally representative level. However, our simulations suggest that adding month-fixed effects in this type of dataset (7 y of data with long, ∼9-mo sampling periods) does provide us with the next best annual estimate that accounts for bias due to seasonality, estimates that we refer to as “month-adjusted.” This month adjustment only measures the average seasonality in wasting associated with each month; it cannot capture the year-to-year variation in true seasons driven by, for instance, changing rainfall or temperature patterns.

We support previous calls to enhance the understanding of how seasonal stresses affect wasting and other measures of nutritional status and to collect data that enables a better understanding of seasonal wasting patterns [9]. Given that patterns of seasonal wasting and their causes differ between countries, such data can help to inform seasonal actions to prevent increases in wasting as well as efforts to adjust national trend estimates in situations where surveys are not administered at the same time each year. To help facilitate interpretation, we also recommend adding dates of data collection and 95% CIs to dashboards being used to track wasting, and when available, information about the seasonality of wasting to facilitate proper interpretation.

## Author contributions

The authors’ responsibilities were as follows – KSS, LB, RAH, ALTL: designed the research; KSS, LBB: analyzed data; KSS, KB, ALTL: wrote the paper; KSS: had primary responsibility for final content; and all authors: read and approved the final manuscript.

## Funding

This work was supported by the Bill & Melinda Gates Foundation through the DataDENT initiative (Grant No. INV-007332), Seattle, WA; the Feed the Future Innovation Lab for Nutrition, which is funded by the United States Agency for International Development under grant number AID-OAA-L-1-00006; and the Feed the Future Food Systems for Nutrition Innovation Lab at Tufts University, cooperative agreement no. 7200AA21LE0001. The funders did not play a role in the design, implementation, analysis, or interpretation of the data.

## Data availability

Data described in the manuscript is publicly and freely available without restriction from the DHS Program.

## Conflict of interest

RAH reports financial support provided by Bill & Melinda Gates Foundation. ALTL reports financial support provided by USAID. All other authors report no conflicts of interest.
