# Supplementary material for: How Does Survey Timing Influence Apparent Wasting Trends? A Case Study from Senegal
Source: Curr Dev Nutr. 2024 Oct 18;10(3):104480. doi: 10.1016/j.cdnut.2024.104480 (PMC13080451; doi:10.1016/j.cdnut.2024.104480)
Supplement: multimedia component 1 [file mmc1.docx]

**Title:** How does survey timing influence apparent wasting trends? A case study from Senegal

**Authors:** Karan S. Shakya^1^, Leah Bevis^1^, Rebecca A. Heidkamp^2^, Andrew L. Thorne-Lyman^2^*

**Supplementary Materials**

**Supplementary Table 1.** Number of observations per month for each survey years.

**Supplementary Table 2.** Percent of children with flagged or missing weight-for-height z-score across survey years.

|  | **Weight-for-height z-score** | | | |  |
| --- | --- | --- | --- | --- | --- |
|  |  | Flagged (%) | Missing (%) | Total Dropped (%) |  |
|  | 2013 | 2.64 | 11.64 | 14.28 |  |
|  | 2014 | 1.53 | 11.02 | 12.55 |  |
|  | 2015 | 1.98 | 10.19 | 12.77 |  |
|  | 2016 | 1.26 | 10.26 | 11.52 |  |
|  | 2017 | 0.33 | 10.96 | 11.29 |  |
|  | 2018 | 0.52 | 8.88 | 9.4 |  |
|  | 2019 | 0.43 | 9.16 | 9.59 |  |
|  | Flagged indicates observations flagged by the DHS for being invalid. | | | |  |
|  | Missing indicates observations with no WHZ values. | | | |  |
|  |  |  |  |  |  |

**Supplementary Table 3.** Regressions that generated the raw (col 1) and month-adjusted (col 2) wasting prevalences estimates

|  |  | Wasting Rates | |  |
| --- | --- | --- | --- | --- |
|  |  | Unadjusted | Adjusted |  |
|  |  |  |  |  |
|  | 2014 | -2.594*** | -3.266*** |  |
|  |  | (0.704) | (0.730) |  |
|  | 2015 | -0.759 | -1.594** |  |
|  |  | (0.700) | (0.750) |  |
|  | 2016 | -1.280* | -2.233*** |  |
|  |  | (0.717) | (0.720) |  |
|  | 2017 | 0.405 | -0.802 |  |
|  |  | (0.638) | (0.672) |  |
|  | 2018 | -0.712 | -2.286*** |  |
|  |  | (0.738) | (0.786) |  |
|  | 2019 | -0.634 | -1.832** |  |
|  |  | (0.738) | (0.802) |  |
|  | Feb |  | 3.544*** |  |
|  |  |  | (1.321) |  |
|  | Mar |  | 3.229*** |  |
|  |  |  | (1.209) |  |
|  | Apr |  | 2.912** |  |
|  |  |  | (1.182) |  |
|  | May |  | 3.441*** |  |
|  |  |  | (1.140) |  |
|  | Jun |  | 4.171*** |  |
|  |  |  | (1.232) |  |
|  | Jul |  | 5.612*** |  |
|  |  |  | (1.221) |  |
|  | Aug |  | 4.183*** |  |
|  |  |  | (1.173) |  |
|  | Sep |  | 4.640*** |  |
|  |  |  | (1.170) |  |
|  | Oct |  | 5.965*** |  |
|  |  |  | (1.116) |  |
|  | Nov |  | 3.854*** |  |
|  |  |  | (1.188) |  |
|  | Dec |  | 3.450*** |  |
|  |  |  | (1.273) |  |
|  | Constant | 8.606*** | 5.375*** |  |
|  |  | (0.511) | (0.896) |  |
|  |  |  |  |  |
|  | Observations | 46,137 | 46,137 |  |
|  | R-squared | 0.001 | 0.003 |  |
|  | F | 4.471 | 4.112 |  |
|  | Standard errors in parenthesis | | |  |
|  | * *p* < 0.10, ** *p* < 0.05, *** *p* < 0.01 | | |  |

|  |  |  |  |  |  |  |  |  |  |  |  |  | |  | |  | |  | |  |
| --- | --- | --- | --- | --- | --- | --- | --- | --- | --- | --- | --- | --- | --- | --- | --- | --- | --- | --- | --- | --- |
|  | **Mali** | | | | | | | | | | | | | | | |  | |  | |
|  |  | *Month* | | | | | | | | | | | | |  | |  | |  | |
|  |  | 1 | 2 | 4 | 5 | 6 | 7 | 8 | 9 | 10 | 11 | 12 | | **Total** | |  | |  | |  |
|  | 2006 | 0 | 0 | 518 | 171 | 3236 | 2911 | 3298 | 2502 | 934 | 472 | 196 | | 14238 | |  | |  | |  |
|  | 2013 | 3679 | 36 | 0 | 0 | 0 | 0 | 0 | 0 | 0 | 2171 | 4440 | | 10326 | |  | |  | |  |
|  | 2018 | 0 | 0 | 0 | 0 | 0 | 0 | 2963 | 4084 | 2590 | 303 | 0 | | 9940 | |  | |  | |  |
|  | **Total** | 3679 | 36 | 518 | 171 | 3236 | 2911 | 6261 | 6586 | 3524 | 2946 | 4636 | | 34504 | |  | |  | |  |
|  |  |  |  |  |  |  |  |  |  |  |  |  | |  | |  | |  | |  |
|  | **Liberia** | | | | | | | | | | | | | |  | |  | |  | |
|  |  | *Month* | | | | | | | | | | |  | |  | |  | |  | |
|  |  | 1 | 2 | 3 | 4 | 5 | 6 | 7 | 10 | 11 | 12 | **Total** | |  | |  | |  | |  |
|  | 2007 | 1876 | 1799 | 1193 | 486 | 0 | 0 | 0 | 0 | 0 | 445 | 5799 | |  | |  | |  | |  |
|  | 2013 | 0 | 0 | 2024 | 2182 | 1366 | 1760 | 274 | 0 | 0 | 0 | 7606 | |  | |  | |  | |  |
|  | 2020 | 2217 | 216 | 0 | 0 | 0 | 0 | 0 | 866 | 1420 | 985 | 5704 | |  | |  | |  | |  |
|  | **Total** | 4093 | 2015 | 3217 | 2668 | 1366 | 1760 | 274 | 866 | 1420 | 1430 | 19109 | |  | |  | |  | |  |
|  |  |  |  |  |  |  |  |  |  |  |  |  | |  | |  | |  | |  |
|  | **Guinea** | | | | | | | | | | | |  | |  | |  | |  | |
|  |  | *Month* | | | | | | | | |  | |  | |  | |  | |  | |
|  |  | 2 | 3 | 4 | 5 | 6 | 7 | 8 | 9 | 10 | **Total** |  | |  | |  | |  | |  |
|  | 2005 | 504 | 1973 | 1722 | 1723 | 442 | 0 | 0 | 0 | 0 | 6364 |  | |  | |  | |  | |  |
|  | 2012 | 0 | 0 | 0 | 0 | 794 | 1756 | 2141 | 2144 | 204 | 7039 |  | |  | |  | |  | |  |
|  | 2018 | 0 | 256 | 3096 | 4327 | 272 | 0 | 0 | 0 | 0 | 7951 |  | |  | |  | |  | |  |
|  | **Total** | 504 | 2229 | 4818 | 6050 | 1508 | 1756 | 2141 | 2144 | 204 | 21354 |  | |  | |  | |  | |  |
|  |  |  |  |  |  |  |  |  |  |  |  |  | |  | |  | |  | |  |
|  | **Nigeria** | | | | | | | | | | | | | | | |  | |  | |
|  |  | *Month* | | | | | | | | | | | | |  | |  | |  | |
|  |  | 2 | 3 | 4 | 5 | 6 | 7 | 8 | 9 | 10 | 11 | 12 | | **Total** | |  | |  | |  |
|  | 2008 | 0 | 0 | 0 | 0 | 2947 | 8004 | 8240 | 5741 | 3673 | 42 | 0 | | 28647 | |  | |  | |  |
|  | 2013 | 1795 | 8801 | 9673 | 9933 | 1168 | 112 | 0 | 0 | 0 | 0 | 0 | | 31482 | |  | |  | |  |
|  | 2018 | 0 | 0 | 0 | 0 | 0 | 0 | 2458 | 9539 | 8420 | 7662 | 5845 | | 33924 | |  | |  | |  |
|  | **Total** | 1795 | 8801 | 9673 | 9933 | 4115 | 8116 | 10698 | 15280 | 12093 | 7704 | 5845 | | 94053 | |  | |  | |  |
|  |  |  |  |  |  |  |  |  |  |  |  |  | |  | |  | |  | |  |

**Supplementary Table 4.** Survey observations across year and month for select countries in Sub-Saharan Africa.


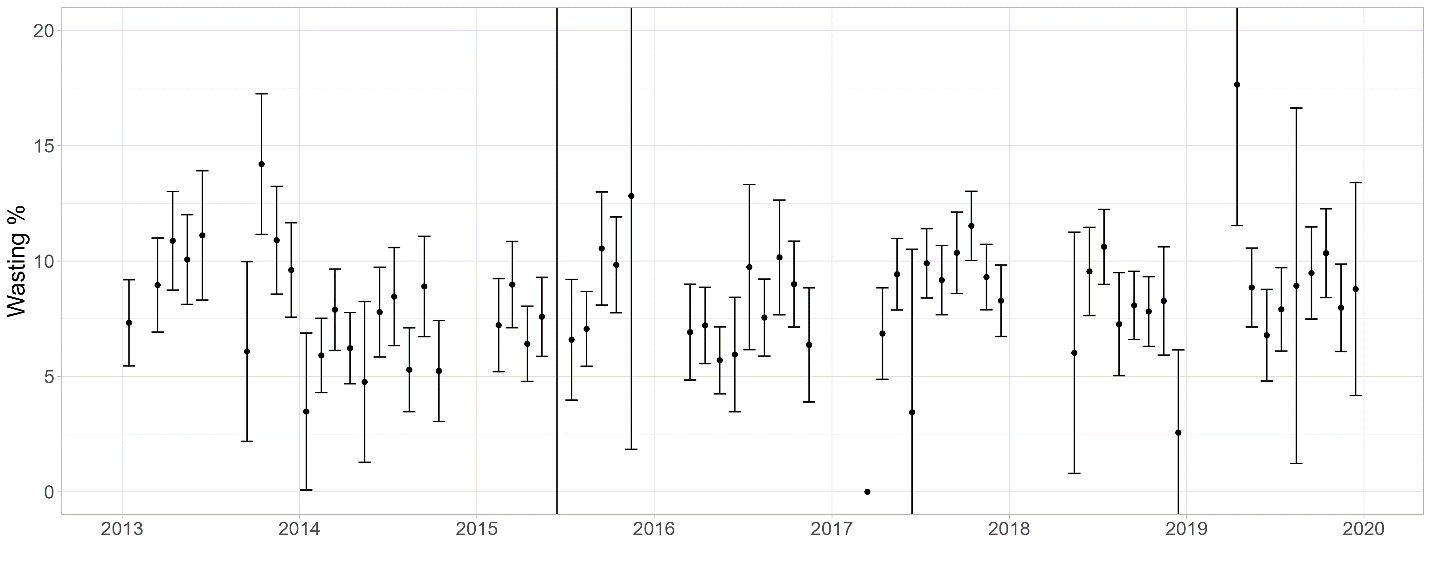
 **Supplementary Figure 1.** Average monthly wasting prevalence across years.

**Supplementary Figure 2.** Simulated, true wasting prevalences in each year (black solid for all-year prevalence, gray dashes for prevalence in the first and second half of the year) compared to estimated wasting prevalence with (green) and without (orange) month fixed effects in a sampling scenario where each year-specific sample is 9 months long and placed randomly within the year. Panels (a) and (b) stem from different simulated, year-round datasets.

**a.**


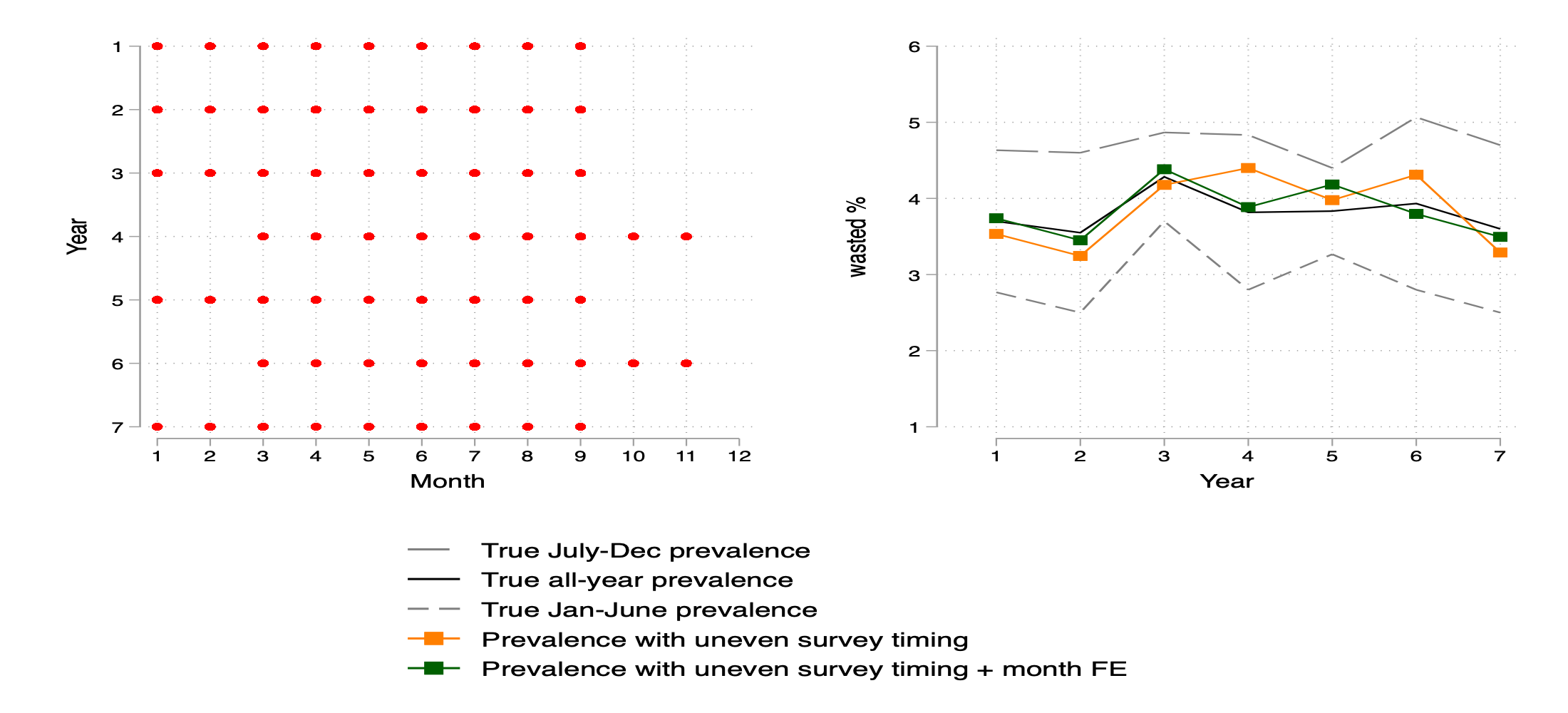


**b.**


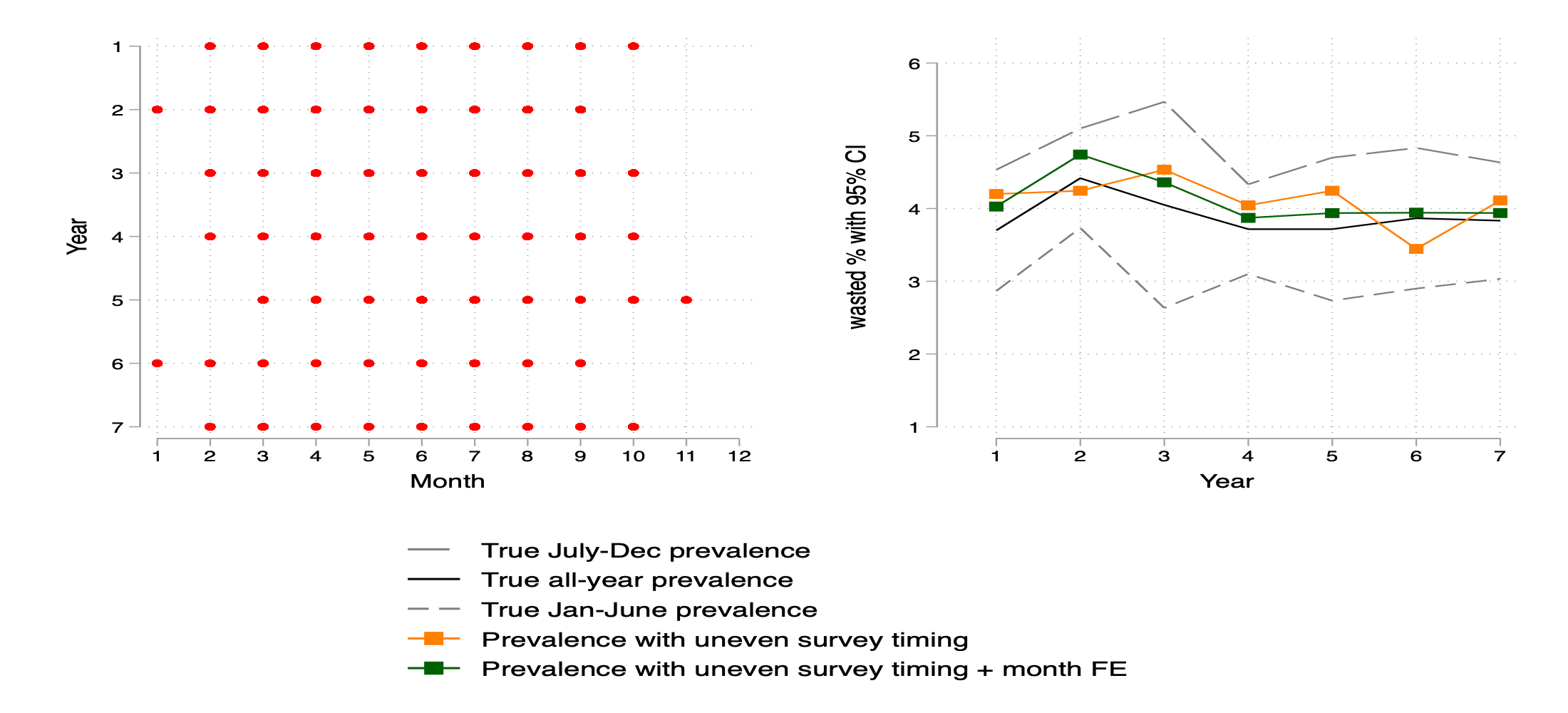


**Supplementary Figure 3** Simulated, true wasting prevalences in each year (black solid for all-year prevalence, gray dashes for prevalence in the first and second half of the year) compared to estimated wasting prevalence with (green) and without (orange) month fixed effects in three sampling scenarios: (a) all 7 surveys conducted within the first half of each year, (b) three surveys beginning as late as May-July, (c) three surveys beginning as late as August-September. Each sampling scenario stems from the same simulated, year-round dataset.

**a.**

**b.**
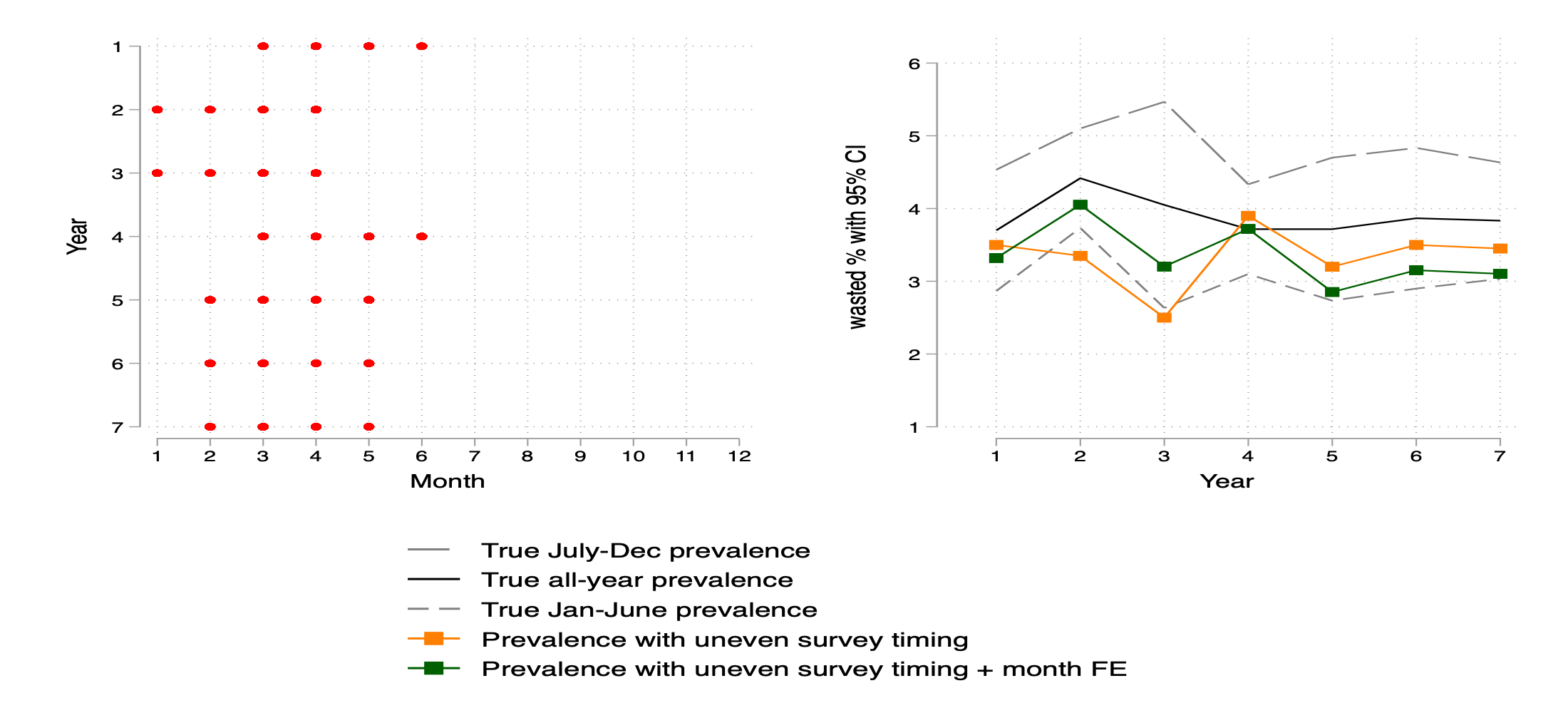

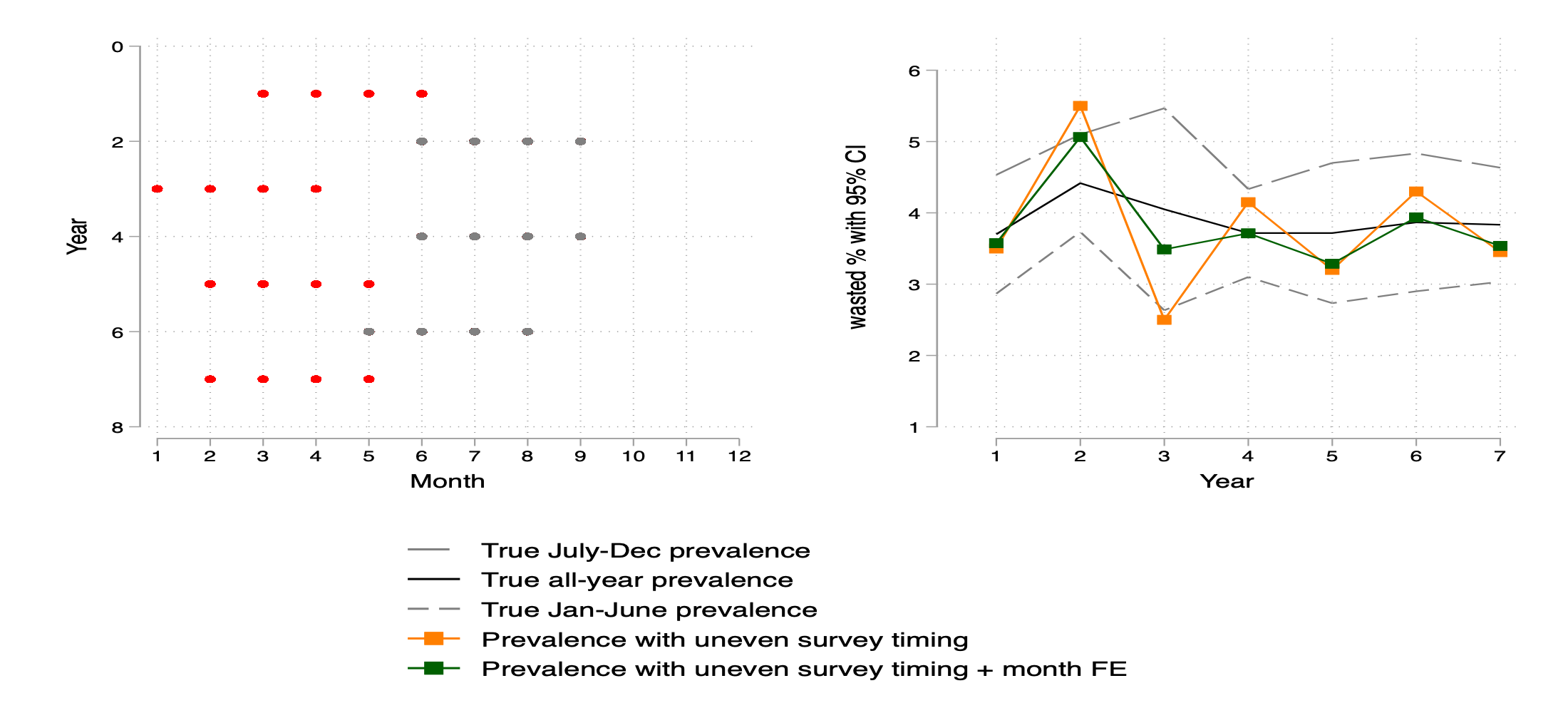


**c.
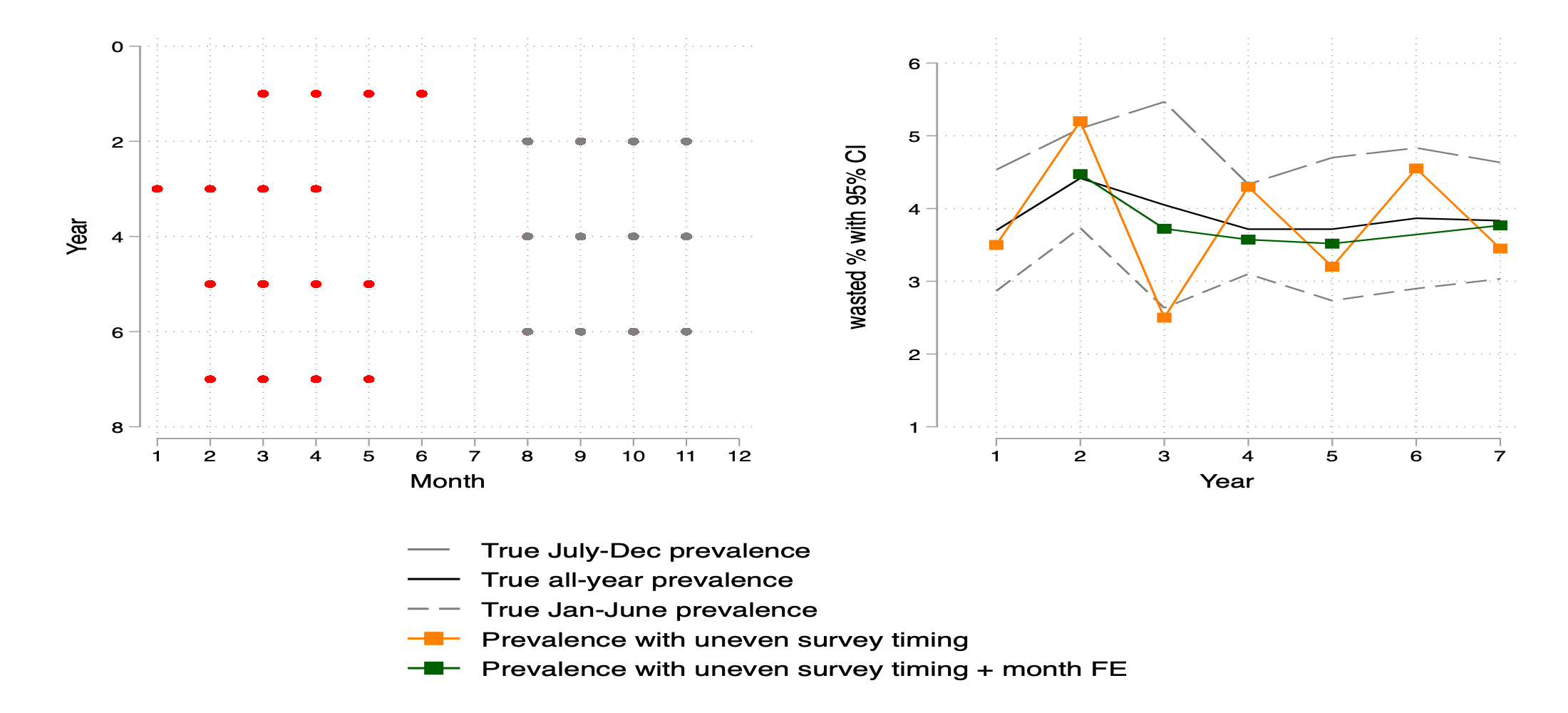
**
